# Nasal reconstruction on supernumerary nostril: A rare case report

**DOI:** 10.1016/j.ijscr.2024.109627

**Published:** 2024-04-09

**Authors:** Irra Rubianti Widarda, Hardisiswo Soedjana, Lisa Y. Hasibuan, Ratnanggana Sistaparamita Lukito

**Affiliations:** Division of Plastic Reconstructive and Aesthetic Surgery, Department of Surgery, Faculty of Medicine, Universitas Padjadjaran, Jalan (street) Prof. Dr. Eyckman 38, Bandung, West Java, Indonesia 40161

**Keywords:** Case report congenital, Nose, Nostril, Reconstruction, Supernumerary

## Abstract

**Introduction:**

A supernumerary nostril is one of the rarest congenital nasal abnormalities. Recently we encountered a 16-months-old patient with 3 supernumerary nostrils that was located at left side of the nose and performed an operation on this case. We reported a rare case of three supernumerary nostril located at left side of the nose.

**Case presentation:**

A 14-month-old male baby was referred to our clinic for assessment of a nose deformity. Physical examination revealed an asymmetrical ala of nose with 3 supernumerary nostrils at left side and a normal right nostril. She was diagnosed with supernumerary rostril and underwent fistulectomy of the accessory nasal tract, followed by nostril reconstruction with primary closure of margin defect at 16-month-old. There was no complication and she was discharged the next day. One month after surgery triamcinolone was injected at scar. Nasal cavity retainer is still maintained in place as aftercare for at least 3-months-post-surgery. We obtained an event-free postoperative result with good function and aesthetic results.

**Discussion:**

Supernumerary nostril is an extremely rare case. It should not be confused with a double nose. Surgery should be performed at an early age. Nevertheless, we performed surgery at 16-month-old baby and still obtained satisfying result.

**Conclusion:**

Surgical technique performed at this rare supernumerary nostril case was fistulectomy of the accessory nasal tract, followed by nostril reconstruction good functions, aesthetic results post operatively.

## Highlights

• Supernumerary nostril is a very rare congenital malformation of the nose.
• It must be differentiated from a double nose.
• Usually, no imaging is required.
• Surgery includes fistulectomy and nasal reconstruction.

## 1 Introduction

A supernumerary nostril is one of the rarest congenital nasal abnormalities, and several cases have been reported in the literature. A supernumerary nostril is an additional nostril that may or may not connect with the regular nasal cavity, sometimes forming a dead-end. It is usually situated above the standard nostril opening, although there has been a reported instance of it being located below the normal nostril. Cases of both one-sided and two-sided supernumerary nostrils have been documented [1].

Supernumerary nostrils exhibit a wide range of variations, making each case particularly fascinating, especially when discussing surgical techniques. This case report will elaborate on a specific instance of a supernumerary nostril, which we successfully managed through surgical intervention.

The presence of supernumerary nostrils, or additional nostrils beyond the normal pair, represents a rare congenital anomaly. The variations in their appearance, location, and whether they communicate with the normal nasal cavity add layers of complexity to their management. These variations can significantly impact the approach and planning of surgical procedures aimed at correcting or removing these extra nostrils [2].

In some cases, a supernumerary nostril may be cosmetically apparent but functionally insignificant, not connecting to the nasal cavity. In others, it may form a cul-de-sac or even have a partial or full connection to the regular nasal pathways, potentially affecting breathing or leading to other complications. The decision to operate, the planning of the surgery, and the surgical techniques employed depend heavily on these factors.

Our case report details a patient with a supernumerary nostril located in an uncommon position, which posed unique challenges for surgical correction. The operation's success was predicated on a thorough understanding of the anomaly's anatomy, precise surgical planning, and the careful execution of the chosen surgical technique.

## 2 Presentation of case

**Fig. 1:**
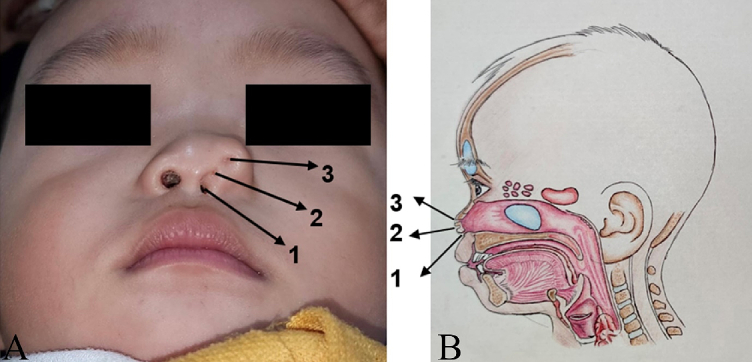
Preoperative view showing 3 supernumerary nostrils at left side of nose from anterior view (A) and illustration from lateral view showing connection between each supernumerary nostrils: nostril number 1 and 3 connected to main ipsilateral nostril, whereas nostril number 2 made a blind end (B).

A fourteen-month-old male toddler was referred to the Plastic Surgery Clinic for assessment of a nose deformity. An event-free pregnancy and born through normal delivery, he nor his family had a history of congenital malformations. Physical examination revealed an asymmetrical ala of nose with 3 supernumerary nostrils at left side (Fig. 1A). The normal right nostril was approximately the same size as the left one. Bony structures were present and there was no evidence of any other malformation. No further cranial imaging was done in this case — we diagnosed this congenital nasal deformity to be supernumerary nostril based on its appearance. We hereby report the case. This report was written according to the SCARE guideline [3].

## 3 Surgical technique

**Fig. 2:**
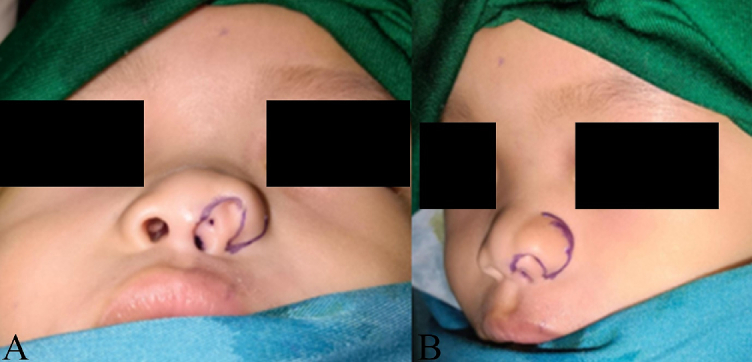
Marking of the incision site and outline of the tract projection from anterior view (A), marking continue alongside the lateral margin of ala of nose from oblique view (B).

The patient underwent surgery at 16 months old. Patient was in supine positon and under general anesthesia. After draping, methylene blue was initially instilled into the supernumerary cavities and it was found that the medial supernumerary nostril (number 1) made a tract connected to main ipsilateral nostril, whereas nostril number 2 made a blind end forming a cul-de-sac with integumentary typical of a normal nose, and the last one (number 3) connected to main ipsilateral nostril (Fig. 1B). The entire area of supernumerary orifices number 2 and 3 with suspected subcutaneous projection was outlined and extended alongside the lateral margin of ala of nose and infiltrated with an anesthetic and vasoconstrictor solution (Fig. 2).

**Fig. 3:**
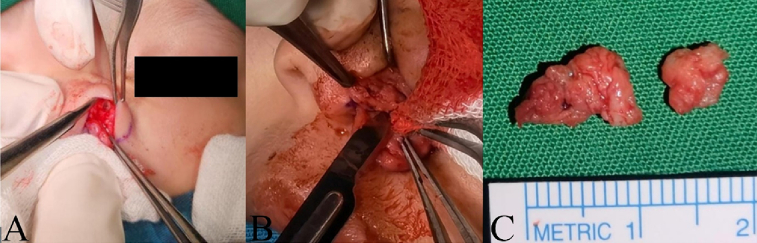
Post lateral supernumerary nostril excision, note the fistular tract opening to ipsilateral nostril (A); post medial supernumerary nostril excision (B); and specimens of supernumerary nostrils (C).

The incision was made around the supernumerary orifices and dissection was made first at number 3 fistular tract up to the opening to main cavity, followed by the entire number 2 fistular tract up to its base (Fig. 3A, B). Surgical specimens at 0.4 and 1.1-cm-long was obtained from supernumerary tracts number 2 and 3, respectively (Fig. 3C). During resection, the supernumerary tracts did not cross path with neither left lateral cartilage nor the medial septum.

**Fig. 4:**
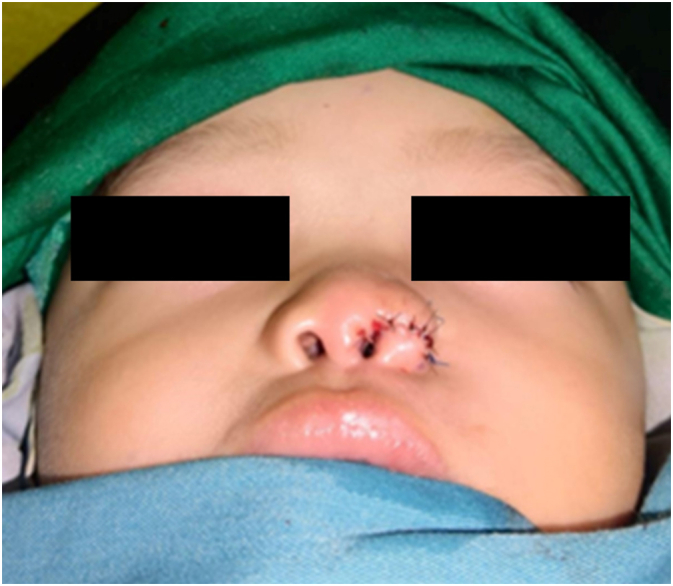
Post surgery.

After fistulectomy for these supernumerary nostrils was completely performed, wound area was shaped into a curved scar running along the nasal rim. The size of contralateral nasal rim was measured for guidance, and excess skin and subcutaneous tissue was resected. Defect was closed with suturing together the wound margins, subcutaneous with PGA 5.0 and skin with nylon 6.0. Chinch sutures were made with prolene 4.0 at 2 points at left alar nose (Fig. 4). Following sutures, nasal retainer was inserted into left nostril and was fixated with PGA 5.0 and hypafix was attached to fixate the PGA 5.0 at glabela.

**Fig. 5:**
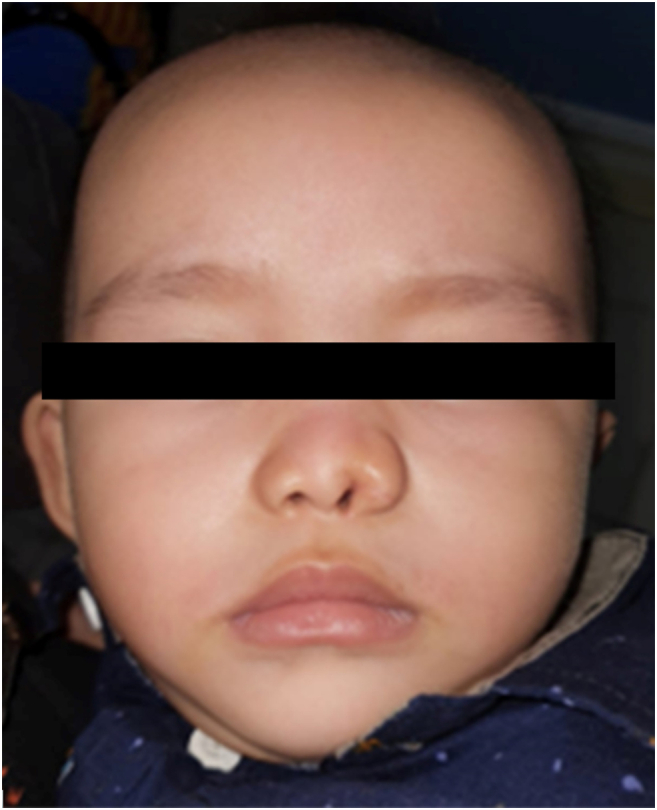
Post-operation-day 1 month.

Sutures were treated with tulle, gauze, and hypafix, and bandage was opened at POD 2 and treated with Chloramphenicol eye ointment 6 times a day. Postoperative recovery was event-free, and the patient was discharged the following day. At 7-day-post-surgery sutures were removed (Fig. 5) and 1-month-post-surgery triamcinolone was injected at scar. Triamcinolone was administered at the site of nasal surgery to mitigate the risk of excessive scar tissue formation. We recognized the critical need to manage scarring effectively, as unchecked scar proliferation could significantly alter the nasal contour and compromise the aesthetic and functional outcomes of the surgery. The inclusion of triamcinolone injection in our treatment protocol aimed to ensure that the scar did not thicken to a degree that would impact the surgery's success, preserving the intended shape and function of the nose post-reconstruction. Nasal cavity retainer is still maintained in place as aftercare for at least 3-months-post-surgery.

## 4 Discussion

We have carefully reviewed the literature to compare our surgical procedure with those described in other published case reports. Our investigation revealed that there are no documented cases that precisely mirror the surgical technique and follow-up approach used in our report. This absence of similar cases underscores the novelty and significance of our case report, highlighting its contribution to the existing body of knowledge on the management of this condition. It appears our surgical approach and the subsequent follow-up results present a unique addition to the medical literature, potentially offering a new perspective on treatment methodologies.

Cases of supernumerary nostril are extremely rare. Lindsay et al. published the first paper in 1906, describing a bilateral supernumerary nostril above normal nostrils, connecting to the ipsilateral nasal pit [4]. Ever since, we found only 48 studies in total reporting cases about supernumerary nostril to this date.

Supernumerary nostril is described as an accessory nostril which may or may not communicate with normal nasal cavity, forming cul-de-sac [5,6]. It is generally located above the normal nostril aperture [7], although a case of supernumerary nostril was reported to be located below normal nostril [8]. Both unilateral [7] and bilateral [4] occurrences have been reported. Franco et al. published a literature review about supernumerary nostrils and described that most cases were found on the left side, above the normal nostril [9].

Supernumerary nostril should not be confused with double nose, defined as a malformation which each nose has its own septum, two well-formed nostrils, and two nasal cavities [7], and with proboscis lateralis, described as a pendulous tubular mass arising superior to the inner canthus, replacing half of the underdeveloped nose [10,11].

Franco et al. in their study conduct non-contrast head CT scan prior, showing projection of the accessory nostril as a cul-de-sac cavity pit separated from the ipsilateral nasal pit by a thin wall of subcutaneous tissue. Prior to surgery methylene blue was instilled into supernumerary nostril and showed no leakage from the ipsilateral nostril, thus confirming the CT findings [9]. This shows no imaging needed thus no further expense needed for showing structures involved since the deformity restricted only at subcutaneous tissue.

Surgery should be performed at an early age, as Franco et al. recommend surgery for supernumerary nostril at three months of age [9]. However, we performed surgery at 16 months old child and still obtain satisfying result without deformation of adjacent structures and no psychological effects that follows.

Saiga et al. in their earlier study classified supernumerary nostril in 4 types based on the shape of the nasus externus and the surgery approach of each type. The most common one is type 1, similar to our case, in which the inner nostril is sufficiently large, and symmetry with the unaffected side can be obtained, and treatment is by resection of the outer nostril [11]. Onizuka et al. in 1972 reported a similar case of supernumerary nostril one above another and the approach performed involves fistulectomy followed by suturing the wound [7]. Another operation of fistulectomy, trimming of redundant skin and primary closure for supernumerary nostrils was reported by Franco et al [9] and Matsumura et al. Fifty years later in our study we still perform the same technique in which relatively simple and still obtained good aesthetical and functional result. Nevertheless, it is important to note that the entire fistular tract must be resected and normal nostril must be preserved or restored [13,14]. Revision of slightly bulky alae of nose in our patient will be carried out around the age of 7–10 years-old to improve symmetricity of nose.

In cases of supernumerary nostril where the greater alar cartilage was under-developed, it was necessary to leave the nasal cavity retainer in place as aftercare [11]. Previous studies suggested for postoperative nasal retainer to be inserted for at least 2–3 months [12,15]. In our study nasal retainer was maintained for at least 4 months to prevent nostril contracture.

Earlier study conducted by Franco et al. stated that nearly half of the patients (45 %) presented with other congenital malformations, and when other facial deformities involves, it might interfere in the surgical treatment selected [7], and more advanced techniques such as nasolabial flaps and grafts [13] might be performed.

## 5 Conclusion

A supernumerary nostril is a rare congenital nasal abnormality. Diagnosis was made based on its appearance and without further cranial imaging, whereas identification of tract and structures involved might be obtained with instilling methylene blue. Surgery was performed at 16-months-old patient, with surgical technique performed was fistulectomy of the accessory nasal tract, followed by nostril reconstruction with primary closure of margin defect at left ala of nose. We obtained good function and aesthetic results regarding postoperative result.

## Financial disclosure statement

There was no any financial disclosure or support for this study.

## Funding

All sources of funding should be declared as an acknowledgement at the end of the text. Authors should declare the role of study sponsors, if any, in the collection, analysis and interpretation of data; in the writing of the manuscript; and in the decision to submit the manuscript for publication. If the study sponsors had no such involvement, the authors should so state.

## Ethical approval

Ethical approval for this study (Ethical Committee No NAC 207) was provided by the Ethical Committee of Dr. Hasan Sadikin General Hospital, Bandung, Indonesia on December 10th, 2023.

## Consent

Written informed consent was obtained from the patient's parents/legal guardian for publication and any accompanying images. A copy of the written consent is available for review by the Editor-in-Chief of this journal on request.

## Declaration of competing interest

All authors must disclose any financial and personal relationships with other people or organisations that could inappropriately influence (bias) their work. Examples of potential conflicts of interest include employment, consultancies, stock ownership, honoraria, paid expert testimony, patent applications/registrations, and grants or other funding.

## References

[1] Choi B.E., Ko S.O., Shin H.K. (2016). Supernumerary nostril: a case report. Maxillofac Plast Reconstr Surg..

[2] Powar R.S., Tubaki V.R. (2007). Supernumerary nostril with complete unilateral cleft lip: a case report and review. The Cleft Palate Craniofacial Journal..

[3] Sohrabi C., Mathew G., Maria N., Kerwan A., Franchi T., Agha R.A. (2023). The SCARE 2023 guideline: updating consensus Surgical CAse REport (SCARE) guidelines. Int J Surg Lond Engl..

[4] Matsumura T., Hayashi A., Komuro Y. (2010). The supernumerary nostril. J. Craniofac. Surg..

[5] Tambwekar S.R., Aiyer P.M., Vij V.K. (1997). Supernumerary nostril in association with incomplete naso-ocular cleft. Plast. Reconstr. Surg..

[6] Sinha R., Das S., Sikder B., Ray S., Bit U.K. (2005). Supernumerary nostril with congenital cataract. Ear Nose Throat J..

[7] Nakamura K., Onizuka T. (1987). A case of supernumerary nostril. Plast. Reconstr. Surg..

[8] Reddy K.A., Rao A.K. (1987). Triple nostrils: a case report and review. Br. J. Plast. Surg..

[9] Franco D., Medeiros J., Faveret P., Franco T. (2008). Supernumerary nostril: case report and review of the literature. J Plast Reconstr Aesthetic Surg..

[10] Abou-Elhamd K.E.A. (2004). Proboscis Lateralis: a report of two cases. Int. J. Pediatr. Otorhinolaryngol..

[11] Kirkpatrick T.J. (1970). Lateral nasal proboscis. J. Laryngol. Otol..

[12] Saiga A., Mitsukawa N. (2013). Case of supernumerary nostril. J Plast Reconstr Aesthetic Surg..

[13] Chen C.T., Chen Y.R. (1994). A case report of triple nostrils. Changgeng Yi Xue Za Zhi.

[14] Deshpande S.N. (1995). Congenital duplication of nostril. Indian J Plast Surg [Internet]..

[15] Mishra L.K., Pradhan S.K., Gupta S., Sahoo S. (2015). Supernumerary nostril: report of a rare case and a review of the literature. Plast Surg Case Stud..

